# Draft genome sequence of *Mycolicibacterium conceptionense* isolated from the sputum sample of a patient with pulmonary tuberculosis

**DOI:** 10.1128/mra.00724-24

**Published:** 2024-10-18

**Authors:** Varsha Chauhan, Anupriya Singh, Rohan Arora, Kamal Shrivastava, Swati Kathait, Sanjay Kumar, Anuj K. Bhatnagar, Mandira Varma-Basil

**Affiliations:** 1 Department of Microbiology, Vallabhbhai Patel Chest Institute, University of Delhi, Delhi, India; 2 Department of Microbiology, Maharshi Dayanand University, Rohtak, India; 3 Department of Chest and Tuberculosis, Rajan Babu Institute of Pulmonary Medicine and Tuberculosis, New Delhi, India; Loyola University Chicago, Chicago, Illinois, USA

**Keywords:** *Mycolicibacterium conceptionense*, nontuberculous mycobacterium, whole-genome sequencing

## Abstract

We report the draft genome of *Mycolicibacterium conceptionense*, a rapidly growing nontuberculous mycobacterium, isolated from the sputum sample of a patient undergoing treatment for multidrug-resistant tuberculosis in Delhi, India. The 6,366,717-bp genome contains 6,124 coding sequences, one 5S rRNA, three 16S rRNAs, six 23S rRNAs, and 49 tRNAs.

## ANNOUNCEMENT

*Mycobacterium conceptionense* (*Mycolicibacterium conceptionense*) is a non-pigmented, rapidly growing nontuberculous mycobacterium (NTM). *M. conceptionense*, first isolated from a case of post-traumatic right tibia osteitis (1), has been observed in both immunocompetent and immunocompromised individuals (2–8).

We report the draft genome sequence of *M. conceptionense*, isolated from the sputum sample of a patient being treated for multidrug-resistant tuberculosis in Delhi, India. The sputum sample was decontaminated, inoculated on Löwenstein-Jensen medium, and incubated at 37°C (9). The organism isolated within 5 days (VP14) was non-pigmented and acid-fast by Ziehl-Neelsen staining and further identified as NTM by duplex PCR (10). Whole-genome sequencing identified the organism to species level.

**Fig 1:**
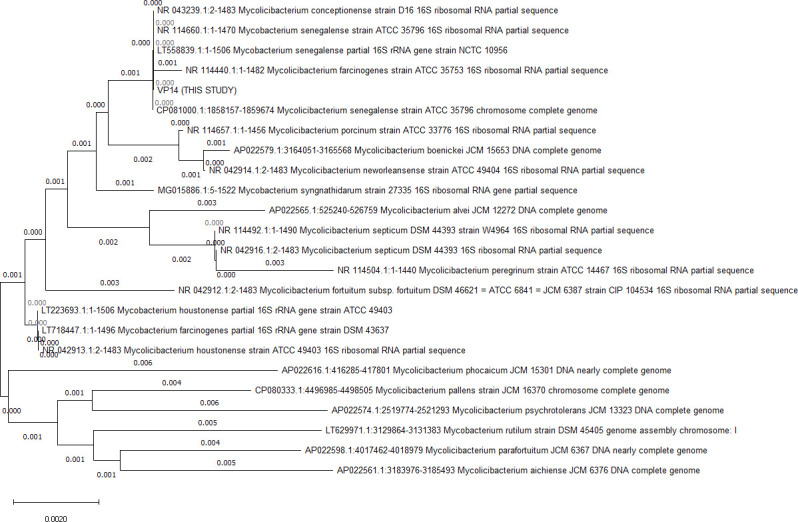
Evolutionary analyses were based on 16S rRNA conducted in MEGA11 after alignment by ClustalW (http://www.ebi.ac.uk/clustalw/). The evolutionary history was inferred using the neighbor-joining method. The optimal tree is shown. The tree is drawn to scale with branch lengths (shown below the branches) in the same units as those of the evolutionary distances used to infer the phylogenetic tree. The evolutionary distances were computed using the Kimura two-parameter method (21) and are in the units of the number of base substitutions per site. This analysis involved 24 nucleotide sequences. Codon positions included were first + second + third + noncoding. All ambiguous positions were removed for each sequence pair (pairwise deletion option). There were a total of 1,522 positions in the final data set.

Genomic DNA was extracted using the cetyltrimethylammonium bromide DNA extraction method (11). Paired-end libraries were prepared using NEBNext Ultra DNA Library Preparation Kit. Sequencing was done on an Illumina NovaSeq 6000 platform. Paired-end reads were quality controlled using FastQC (version 0.12.1) (12). Of the initial 11.628194 reads obtained, 11.556870 (mean length 150 bp) were recovered after filtering and trimming by fastp (version 0.23.2) (13). The genome was assembled using Shovill Faster SPAdes pipeline (version 1.1.0, SPAdes version 3.14.1) (14), resulting in 65 contigs to an assembly of 6,366,717 bp (estimated sequencing depth 274×; coverage 100.147×). Default parameters were used for all software. Annotation was carried out by the NCBI Prokaryotic Genome Annotation Pipeline (version 6.7) (15). The 6,366,717 bp genome contains 6,124 coding sequences, one 5S rRNA, three 16S rRNAs, six 23S rRNAs, and 49 tRNAs. The genome assembly was screened for 16s RNA contamination using ContEst16S (16). 16s RNA evaluated against NCBI rRNA Database matched *M. conceptionense* (NR_043239.1) and *Mycobacterium senegalense* (NR_114660.1) with 100% identity and 97% and 96% query cover, respectively. Other phylogenetic neighbors included *Mycobacterium farcinogenes* (NR_114440.1), *Mycobacterium porcinum* (NR_042920.1)*, Mycobacterium houstonense* (NR_042913.1), and *Mycobacterium neworleansense* (NR_042914.1). A neighbor-joining phylogenetic tree based on *16S RNA* gene was created using Molecular Evolutionary Genetics Analysis version 11 (MEGA11) (Fig. 1) (17), clustered VP14, *M. conceptionense, M. senegalense,* and *M. farcinogenes*. Automated Multi-Locus Species Tree was created using autoMLST in *de novo* mode (18), the closest species identified being *Mycobacterium conceptionense* with 100% average nucleotide identity (ANI). ANI was also calculated with DFAST Quality Control (Taxonomy and Completeness check of the genome) version 1.6.0 (24 March 2022) (19), and the organism was confirmed as *M. conceptionense* (ANI 98.86%) as the widely accepted cutoff adopted for the ANI is 95%–97% (20).

Genome annotation via DFAST suggested that the GC content of the genome was 66.4% (*N*_50_ value 618,141; 0.0% gap ratio; 92.7% coding ratio). Distance Calculator (GGDC) was used through the Type (Strain) Genome Server (https://tygs.dsmz.de) (22). dDDH values of VP14 in comparison with *Mycobacterium conceptionense* CCUG 50187, *Mycobacterium conceptionense* D16, and *Mycobacterium senegalense* DSM 43656 were 97.0% , 89.8%, and 86.0%, respectively, identifying the organism as *M. senegalense*. Our findings aligned with previous reports (23), indicating that *M. senegalense* and *M. conceptionense* were synonymous.

The genome of *M. conceptionense* presented here provides essential data for future phylogenetic and comparative genome studies.

## Data Availability

This Whole Genome Shotgun project has been deposited at DDBJ/ENA/GenBank under the accession JBEUKP000000000. The version described in this paper is version JBEUKP000000000. The BioProject accession number is PRJNA1097974, SRA accession number is SRR28596277, and BioSample is SAMN40892597.
